# Characterising Negative Mental Imagery in Adolescent Social Anxiety

**DOI:** 10.1007/s10608-022-10316-x

**Published:** 2022-07-05

**Authors:** Kenny Chiu, David M. Clark, Eleanor Leigh

**Affiliations:** 1 Department of Clinical Psychology & Psychological Therapies, University of East Anglia, Norwich, UK; 2 Department of Experimental Psychology, University of Oxford, Oxford, UK

**Keywords:** Adolescent, Prospective, Social anxiety, Negative self-imagery, Observer-perspective, Vividness

## Abstract

**Background:**

Understanding the role of self-imagery in the development of social anxiety in adolescence holds promise for improving intervention. Cross-sectional studies indicate that imagery characteristics are associated with social anxiety symptoms, however, prospective studies are lacking. The current study examined concurrent and prospective associations between two image characteristics, namely observer-perspective and vividness, with social anxiety symptoms in a community adolescent sample (*N* = 616; 53% girls; aged 11–15 years). In addition, we examined common themes in the negative social anxiety-related images.

**Methods:**

Negative self-imagery and social anxiety symptoms were assessed using questionnaires at baseline and at 4–6-month follow-up. A series of multiple linear regression analyses were performed to see if each image characteristic predicts concurrent and prospective social anxiety symptoms. Topic modelling was performed to infer key topics from verbal data.

**Results:**

Observer-perspective and vividness significantly predicted concurrent social anxiety symptoms beyond the influence of age and gender. Observer-perspective significantly predicted prospective levels of social anxiety symptoms beyond the influence of age, gender, and baseline social anxiety and depression symptoms. Negative self-images clustered into two themes: the fear of appearing anxious and the fear of being judged or viewed as unacceptable.

**Conclusions:**

Specific characteristics and contents of negative self-images may be particularly relevant to the development of adolescent social anxiety.

**Supplementary Information:**

The online version contains supplementary material available at 10.1007/s10608-022-10316-x.

## Introduction

At the heart of social anxiety disorder (SAD) is the fear of being humiliated or rejected by others. Common concerns for individuals with SAD include “I will blush”, “I will have nothing to say”, and “people will laugh at me”. As well as verbal thoughts, mental images, by which we mean perceptual representations in the mind’s eye that are based on memory rather than available sensory information (Hirsch & Holmes, 2007), are also frequently reported by individuals with SAD (Hackmann et al., 2000). In adult samples, these self-images have been found to be sensorially-laden, excessively negative, and typically seen from an observer-perspective (Dobinson et al., 2020; Hackmann et al., 2000). Examples include picturing oneself dripping in sweat and hearing one’s voice quivering and shaking. In this study we are interested in examining self-imagery in adolescent social anxiety.

Cognitive behavioural accounts emphasise the role of mental imagery (also known as self-imagery) in the maintenance of SAD (Clark & Wells, 1995; Hofmann, 2007; Rapee & Heimberg, 1997). It has been proposed that self-imagery that is excessively negative, vivid, and seen from an observer perspective functions as a maintenance mechanism by directly increasing anxiety, confirming negative self-evaluations, enhancing self-focused attention, and motivating the use of safety behaviours (Clark & Wells, 1995). In a systematic review on self-imagery in social anxiety, Ng et al. (2014) found that imagery valence, perspective, and vividness are the three main imagery characteristics that have been examined in previous studies. Across questionnaire-based and experimental studies, imagery valence and imagery perspective have been consistently found to be relevant to the persistence of adult social anxiety symptoms/disorder. In relation to image valence, three studies that all used similar, robust experimental designs (Hirsch et al., 2003, 2004; Makkar & Grisham, 2011) found that both high and low socially anxious adults endorsed higher self-reported anxiety, more negative self-evaluations, and increased self-focused attention when holding a negative compared to a benign image in mind during a social task. Similarly, in relation to observer perspective, in an experimental study manipulating perspective during a speech task, an observer, compared to a field, perspective was associated with more anxiety, more frequent negative thoughts, and a greater use of safety behaviours (Spurr & Stopa, 2003). Findings regarding imagery vividness have been more mixed. For example, Ng et al. (2014) identified six studies included imagery vividness as an outcome variable. In an early study by Anderson and Borkovec (1980), imagery vividness was assessed on a self-report five-point scale, and the study reported a significant group difference in vividness between negative and control social anxiety-related imagery. However, subsequent studies either showed null or opposite findings (Hirsch et al., 2003; Hulme et al., 2012; Makkar & Grisham, 2011; Moscovitch et al., 2011; Stopa & Jenkins, 2007). Given these inconsistencies across studies, Ng et al. (2014) concluded that further studies examining imagery vividness with robust methodology are needed. Taken together, findings suggest that negative observer-perspective self-imagery may be a valid therapeutic target, whereas findings for vividness are mixed. Indeed, in Cognitive Therapy for SAD (CT-SAD; Clark et al., 2003), negative observer-perspective self-imagery is already addressed using video feedback with verbal preparation (Warnock-Parkes et al., 2017) and imagery rescripting (Wild & Clark, 2011).

Nearly all cases of SAD have their onset in adolescence (Kessler et al., 2005) and so it is important to understand the role of self-images in social anxiety at this time of life. Due to the considerable neurocognitive development occurring during this period (Kilford et al., 2016), including in the ability to generate, inspect, maintain, and manipulate mental images (Burnett Heyes et al., 2013), direct investigation of the nature and role of self-imagery in youth is needed to answer this. In particular, it is important to establish whether the same characteristics of imagery that are relevant to the persistence of adult social anxiety also operate in adolescents.

Most studies that have been carried out with adolescent samples have focused on valence of social anxiety related imagery. In addition to three questionnaire-based studies that have found an association between negatively-valenced imagery and adolescent social anxiety (Ranta et al., 2014; Schreiber & Steil, 2013; Schreiber et al., 2012), two experimental studies have examined this issue. Alfano et al., (2008) recruited adolescents with SAD and healthy controls to take part in a social performance task. Half of the healthy control participants were instructed to engage in negative mental imagery during the task. The remaining healthy controls and youth with SAD were given no instructions. The only differences were that youths with SAD were rated as more anxious and less socially competent than healthy controls in both conditions. However, the lack of a controlled comparison condition, such as a benign imagery condition, may explain the null findings. A recent experimental study with socially anxious adolescents compared two controlled imagery conditions (negative vs. benign) during a conversation task (Leigh et al., 2020). Here, findings were in line with adults: when holding a negative self-image in mind, participants reported more self-reported anxiety and more negative self-evaluations during a conversation task, and participants were rated more critically by blinded independent assessors, compared to when holding a benign image in mind. Taken together, support for the association between negative valence and social anxiety has come from all three of the observational studies and one of the two experimental studies.

We know much less about the importance of observer-perspective and vividness in adolescent social anxiety. All three of the studies examining observer-perspective have found to be associated with adolescent social anxiety (symptoms and disorder) (Hignett & Cartwright-Hatton, 2008; Ranta et al., 2014; Schreiber & Steil, 2013). In the study by Hignett and Cartwright-Hatton (2008), the Perspective-Taking Rating Scale (Wells et al., 1998) was used to measure observer-perspective on a 7-point scale (range of − 3 “field perspective” to + 3 “observer perspective”). Participants were asked to recall their self-image in a social situation and report their ratings. The study by Ranta et al. (2014) used a similar procedure to elicit self-images and participants were asked to report whether their image was from a field- or an observer-perspective. In the study by Schreiber and Steil (2013), they used a self-report questionnaire to explore the characteristics and nature of negative self-imagery in adolescents. One of the questionnaire items assessed observer-perspective on a 0–100 visual analogue scale (range of 0 “field perspective” to 100 “observer perspective”). To date, we found one study that examined the association between vividness and adolescent social anxiety. In the study by Schreiber and Steil (2013), they hypothesised that adolescents with SAD would report more vivid negative self-imagery than the control group. In line with their hypothesis, they found that vividness was higher in a group of adolescents with SAD compared to non-anxious controls (Schreiber & Steil, 2013). However, all the studies examining image perspective and vividness were limited by a cross-sectional design and therefore one cannot draw inferences about the relevance of these characteristics to the persistence of adolescent social anxiety (Chapman et al., 2020; Leigh & Clark, 2018).

Whilst the findings described above are generally consistent with the suggestion that self-imagery is involved in the maintenance of social anxiety in youth in the same way as it is in adults, certain questions remain. First, no studies with youth have yet examined whether observer-perspective and vividness are associated with prospective elevations in social anxiety symptoms. It is critical to understand whether these aspects of self-imagery are implicated in maintenance of social anxiety and therefore a viable target of intervention. Second, little is known about the common themes of negative self-imagery in adolescent social anxiety. A recent qualitative study has investigated mental imagery in anxiety disorders in adolescents using a thematic analysis (Ghita et al., 2021). However, this study asked participants about the characteristics of their mental images rather than the content and was not specifically focused on social anxiety. To our knowledge, no qualitative studies have been conducted to understand the content of these self-images in adolescents. A better understanding of typical themes that occur will allow for more accurate assessment and targeting of problematic imagery.

The present study therefore had two aims. First, to examine concurrent and prospective associations between image characteristics, namely observer-perspective and vividness, with social anxiety symptoms, and second, to identify common themes in the negative social anxiety-related images reported by adolescents. The first hypothesis was that the extent to which imagery was experienced from an observer-perspective would predict concurrent and prospective social anxiety symptoms. Given the mixed findings in relation to vividness and social anxiety in adults, no specific hypothesis was made about self-reported imagery vividness and adolescent social anxiety symptoms. In this prospective study, adolescents completed measures of social anxiety and depression symptoms and a measure of social anxiety-related self-imagery at baseline. They also completed the social anxiety symptom measure again at 4–6-month follow-up. As social anxiety symptoms tend to vary continuously across the population (Stopa & Clark, 2001), the use of an unselected school sample offers a valid and pragmatic approach to examining social anxiety-related phenomena. At baseline, adolescents were asked to describe their self-images in words. These words were recorded on the database. An unsupervised machine learning approach called Latent Dirichlet Allocation (Blei et al., 2003) was used to model latent topics from these words based on word distribution and word co-occurrence. This novel approach is preferred over traditional qualitative approaches, such as thematic analysis, as it can be used to analyse unstructured verbal information automatically, systematically, and statistically. This computational approach has been widely adopted to infer key topics from large text datasets in public health, psychology, and medical research (Feldhege et al., 2020; Geletta et al., 2019; Xue et al., 2020).

## Methods

### Participants and Procedures

Participants aged 11–15 years were recruited from two mainstream secondary schools in London, UK. Written informed assent was obtained from participants and opt-out consent was provided by their parents. The Central University Research Ethics Committee at the University of Oxford approved the study procedures (Reference number: R54283/RE001). A total of 616 students in school years 7–9 were invited to participate in this study. The study sample consisted of 616 participants (53% females) aged 11–15 years (mean = 12.97, SD 0.87) who responded to a questionnaire pack evaluating social anxiety symptoms, depression symptoms and self-image at Time 1. 330 participants completed the questionnaires at Time 1 (54% completion rate). Four to 6 months later (Time 2), 240 participants completed another questionnaire pack that measured social anxiety symptoms. None of the participants had difficulty reading or understanding English. Demographic data was consistent with national statistics (UK Statistics Authority, 2020). 13% of the students were eligible for free school meals, for 13% English was not their first language, and 12% have special educational needs.

### Measures

The Questionnaire of Recurrent Images for Social Phobia (QRI-SP; Schreiber et al., 2009) was used to assess the content, characteristics, emotions, sensations, and associated memory of self-images in adolescents (see online Appendix A1). The original version of this questionnaire was developed for use with adolescents in Germany (Schreiber et al., 2012). This 12-item questionnaire was translated and back-translated by two psychology postgraduate students with German as their first language (LN and SN). The translated version was reviewed by the authors (KC, EL, and DMC).

The first two items assess the *content* of the self-image. In Item 1, participants were asked to describe a self-image they experience in social situations when they felt they looked awkward, foolish, or anxious. Their descriptions were screened by two assessors (KC and EL) independently. A variable called “negative image” was created to indicate the presence of a description of the content of a negative self-image (presence = 1, absence = 0). Spurious entries (e.g., “I’m lying in a hammock”, “I don’t understand this question”) were coded as “absence”. Images that were positive or neutral in content were excluded. Disagreement on coding was discussed and final decisions were recorded on the dataset. The inter-rater reliability was α = 0.89. When it was unclear whether the description was an image or a past memory, the description was coded as “presence” to avoid potential loss of data. In Item 2, eight themes commonly reported by adults with SAD (Hackmann et al., 2000) are presented (e.g., “I get into a situation in which I am judged negatively by others”). Participants were asked to indicate the extent to which their self-image relates to each theme on a 0–100 visual analogue scale (from ‘*not at all*’ to ‘*extremely*’). Items 3–8 assess the *characteristics* of self-image and co-occurring emotions and sensations. Item 3 assesses the frequency of co-occurring emotions on a 0–100 visual analogue scale (e.g., shame, anxious, embarrassed, angry, stupid, insecure, nervous, vulnerable, humiliated/ridiculous; from ‘not at all’ to ‘extremely’). Next, participants rated the level of distress (Item 4) and vividness (Item 5, e.g. “How clear/vivid is this image for you?”) associated with their self-image. They also rated the frequency of their self-image within the last 14 days (Item 6) and the frequency of co-occurring sensations (Item 7) from 1 (*never*) to 5 (*very often*). Item 8 assesses one’s perspective in the self-image. Participants were asked to indicate if they see themselves through their own eyes (self-perspective) or other people’s eyes (observer-perspective) on a 0–100 visual analogue scale. The remaining four items assess the presence, content, and characteristics of a memory associated with one’s self-image. Participants were asked to report if they had a relevant past memory by answering “yes” or “no”. For those who selected “yes”, they were asked to describe their memory in a few words and rate the negativity and vividness on a 0–100 visual analogue scale. The observer-perspective (Item 8) and vividness (Item 5) scores were selected as predictor variables in this study.

Social anxiety symptoms were measured using the Liebowitz Social Anxiety Scale for Children and Adolescents-Self Report version (LSAS-CA-SR; Masia-Warner et al., 2003). This 24-item self-report scale assesses levels of fear and avoidance in social and performance situations in youth aged 7–18 years old on a scale from 0 to 3. The total score was obtained by adding up the 48 items. The self-report version of the scale displays good reliability and validity (Leigh & Clark, 2021a, 2021b). The internal consistency in this sample was α = 0.96 (Time 1) and α = 0.97 (Time 2).

Depressive symptoms were assessed with the Short Mood and Feelings Questionnaire (SMFQ; Angold et al., 1995), which is a 13-item self-report questionnaire designed to assess symptoms in youth aged 6–17 years. Each item ranges from 0 (*not true*) to 2 (*true*). Total score was obtained by summing up all the items. This scale has good reliability and validity (Angold et al., 1995). The internal consistency in this sample was α = 0.92 (Time 1).

### Data Analysis Plan

Statistical analyses were performed using R version 4.0.0 (R Core Team, 2020). When less than 10% of items were missing in each questionnaire, mean substitution was performed. Questionnaires with more than 10% of missing items were treated as missing variables.

We conducted further data analyses to understand the nature of data missingness. Little’s MCAR tests indicated that data was not missing completely at random (MCAR; *ps* < 0.05) in each regression analysis. Regression analyses found that participants who reported older ages and higher levels of social anxiety and depressive symptoms are more likely to complete the questionnaires both at Time 1 and Time 2 (*ps* < 0.05). These results supported the idea that the data is missing at random. Given these findings, we used multiple imputation to handle missing data before running regression analyses because complete case analysis can bias estimates of means, regression coefficients, and correlations (Little & Rubin, 2002; Schafer & Graham, 2002). Results obtained from using complete case analysis are available in online Appendix (see online Appendix A2 Table 3). When outliers were identified using Mahalanobis distance and excluded from the dataset, the same results were obtained, and so the results of the full dataset are presented here. Pearson’s correlational analyses were performed for the study variables (i.e., age, social anxiety symptoms, depressive symptoms, vividness, and perspective-taking). A series of multiple linear regression analyses were performed to see if each image characteristic (observer-perspective and vividness) predicts concurrent social anxiety symptoms, above and beyond the effects of age and gender, with and without controlling for baseline social anxiety and/or depressive symptoms.

Latent Dirichlet Allocation (LDA) was performed to infer key topics in a subset of participants who reported and provided a written description of a negative self-image (*n* = 131). First, a document-term-matrix that describes the frequency of occurrence of each term in each participant was created. Single words (unigrams) as well as word pairs (bigrams) were extracted. Less meaningful words (e.g., “the”, “he”, “she”, “a”) were removed by screening two standard English dictionaries (they are called “en” and “smart”) from the textmineR package (Jones & Doane, 2019). Punctuations, numbers, and suffixes from a word were removed. All words were lowercased and reduced to their word stem (e.g., ‘friendship’ and ‘friends’ were reduced to ‘friend’). Next, LDA was applied to the document-term matrix to cluster words that tend to occur together. To determine the optimal number of topics, multiple LDA models were created by increasing the number of topics (k) from 1 to 100. Each model has an average coherence value, which refers to the average strength of association between words under the same topic. The higher the average coherence value, the better the topic quality. The top terms and word clouds of each topic were presented in the results section.

## Results

### Correlations Between Study Variables

Correlational analyses were performed to examine the strengths of associations between study variables. Table 1 shows that age was positively and significantly correlated with social anxiety symptoms, depressive symptoms, vividness, and observer-perspective. Therefore, it was controlled for in subsequent regression analyses predicting social anxiety symptoms. Table 2 shows that Time 1 social anxiety and depressive symptoms were significantly and positively correlated with Time 2 social anxiety symptoms, and they were controlled for in regression analyses predicting prospective social anxiety symptoms, in line with our a priori decision.

**Table 1:** Pearson’s correlations between study variables measured at Time 1 (N = 616)

Variable	*M*	*SD*	1	2	3	4
1. Age (Time 1)	12.97	0.87				
2. Social anxiety symptoms (Time 1)	41.00	28.69	0.19** [0.17, 0.21]			
3. Depressive symptoms (Time 1)	7.10	6.43	0.18** [0.16, 0.20]	0.59** [0.58, 0.60]		
4.Vividness (Time 1)	44.54	34.97	0.08** [− 0.06, 0.10]	0.31** [0.29, 0.33]	0.36** [0.35, 0.38]	
5. Observer-perspective (Time 1)	41.86	35.99	0.19* [0.17, 0.21]	0.30** [0.29, 0.32]	0.25** [0.24, 0.27]	0.17** [0.15, 0.19]

Descriptive statistics and correlations of imputed variables are presented. *M* and *SD* are used to represent mean and standard deviation, respectively. Values in square brackets indicate the 95% confidence interval for each correlation. The confidence interval is a plausible range of population correlations that could have caused the sample correlation (Cumming, 2014)

*Indicates *p* < 0.05

**Indicates *p* < 0.01

**Table 2:** Pearson’s correlations between study variables measured at Time 1 and Time 2 (*N* = 616)

Variable	*M*	*SD*	1	2	3	4	5
1. Age (Time 1)	12.96	0.87					
2. Social anxiety symptoms (Time 1)	40.92	28.63	0.19* [0.17, 0.21]				
3. Depressive symptoms (Time 1)	7.06	6.38	0.17** [0.15, 0.19]	0.59** [0.57, 0.60]			
4. Social anxiety symptoms (Time 2)	39.51	30.05	0.14** [0.12, 0.16]	0.75** [0.74, 0.76]	0.54** [0.53, 0.55]		
5. Vividness (Time 1)	44.61	34.94	0.09** [0.06, 0.11]	0.30** [0.28, 0.32]	0.35** [0.33, 0.37]	0.24** [0.22, 0.26]	
6. Observer-perspective (Time 1)	42.27	36.08	0.17** [0.15, 0.19]	0.29** [0.27, 0.31]	0.26** [0.24, 0.28]	0.32** [0.30, 0.33]	0.28** [0.16, 0.20]

Descriptive statistics and correlations of imputed variables are presented. *M* and *SD* are used to represent mean and standard deviation, respectively. Values in square brackets indicate the 95% confidence interval for each correlation. The confidence interval is a plausible range of population correlations that could have caused the sample correlation (Cumming, 2014)

*Indicates *p* < 0.05

**Indicates *p* < 0.01

### Image Characteristics Predict Concurrent and Prospective Social Anxiety Symptoms

Regression analysis demonstrated that observer-perspective significantly predicted concurrent social anxiety symptoms, above and beyond the effects of age and gender (*p* < 0.001) and the effect remained significant after controlling for baseline depressive symptoms (*p* < 0.01) (see Table 3). Observer-perspective significantly predicted prospective social anxiety symptoms, above and beyond the effects of age, gender, and baseline social anxiety symptoms (*p* < 0.05), and the effect remained significant (*p* < 0.05) when additionally controlling for baseline depressive symptoms (see Table 4).

**Table 3:** Results of multiple regression analysis for observer-perspective predicting Time 1 social anxiety symptoms (*N* = 616)

Variable	Model 1^1^				Model 2^2^			
	β (SE)	t	*p*	*R*^2^	β (SE)	t	*p*	*R*^2^
*Step 1*				0.08				0.36
Age	0.18 (0.04)	4.53	< 0.001*		0.08 (0.03)	2.50	< 0.05*	
Gender (male)	− 0.44 (0.08)	− 5.65	< 0.001*		− 0.12 (0.07)	− 1.74	0.08	
Depression					0.56 (0.05)	15.30	< 0.001*	
*Step 2*				0.16				0.37
Age	0.13 (0.04)	3.19	< 0.01*		0.06 (0.03)	1.79	0.07	
Gender (male)	− 0.45 (0.08)	− 5.61	< 0.001*		− 0.13 (0.07)	− 2.14	0.03*	
Depression					0.51 (0.04)	12.93	< 0.001*	
Observer-perspective	0.28 (0.05)	5.33	< 0.001*		0.16 (0.05)	3.22	< 0.01*	

*β* standardised beta coefficient, *SE* standard error

*Indicates *p* < 0.05

^1^Model 1 controlled for age and gender

^2^Model 2 controlled for age, gender, and Time 1 depressive symptoms

**Table 4:** Results of multiple regression analysis for observer-perspective predicting Time 2 social anxiety symptoms (*N* = 616)

Variable	Model 1^1^				Model 2^2^			
	β (SE)	t	*p*	*R*^2^	β (SE)	t	*p*	*R*^2^
*Step 1*				0.56				0.58
Age	− 0.01 (0.03)	− 0.15	0.88		− 0.02 (0.03)	− 0.44	0.66	
Gender (male)	− 0.08 (0.06)	− 1.31	0.19		− 0.03 (0.06)	− 0.50	0.61	
Social anxiety	0.74 (0.03)	23.81	< 0.001*		0.66 (0.04)	17.55	< 0.001*	
Depression					0.15 (0.04)	3.47	< 0.001*	
*Step 2*				0.57				0.59
Age	− 0.02 (0.04)	− 0.52	0.60		− 0.03 (0.03)	− 0.74	0.46	
Gender (male)	− 0.10 (0.06)	− 1.62	0.11		− 0.05 (0.06)	− 0.84	0.39	
Social anxiety	0.71 (0.03)	22.26	< 0.001*		0.64 (0.04)	16.60	< 0.001*	
Depression					0.14 (0.04)	3.20	0.01*	
Observer-perspective	0.11 (0.04)	2.90	0.01*		0.10 (0.04)	2.59	0.01*	

*β* standardised beta coefficient, *SE* standard error

^1^Model 1 controlled for age, gender, and Time 1 social anxiety symptoms

^2^Model 2 controlled for age, gender, Time 1 social anxiety symptoms, and Time 1 depressive symptoms

*Indicates *p* < 0.05

Regression analysis showed that vividness significantly predicted concurrent social anxiety symptoms, above and beyond the effects of age and gender (*p* < 0.001) and the effect remained significant after controlling for baseline depressive symptoms (*p* < 0.01) (see Table 5). Vividness did not significantly predict prospective social anxiety symptoms, above and beyond the effects of age, gender, and baseline social anxiety symptoms, or above baseline depressive symptoms (*ps* > 0.05) (see Table 6).

**Table 5:** Results of multiple regression analysis for vividness predicting Time 1 social anxiety symptoms (*N* = 616)

Variable	Model 1^1^				Model 2^2^			
	β (SE)	t	*p*	*R*^2^	β (SE)	t	*p*	*R*^2^
*Step 1*				0.08				0.36
Age	0.18 (0.04)	4.53	< 0.001*		0.08 (0.03)	2.50	< 0.05*	
Gender (male)	− 0.44 (0.08)	− 5.65	< 0.001*		− 0.12 (0.07)	− 1.74	0.08	
Depression					0.56 (0.05)	15.30	< 0.001*	
*Step 2*				0.16				0.37
Age	0.16 (0.04)	4.02	< 0.001*		0.08 (0.03)	2.50	< 0.05*	
Gender (male)	− 0.39 (0.08)	− 5.09	< 0.001*		− 0.13 (0.07)	− 1.18	0.07	
Depression					0.51 (0.04)	12.78	< 0.001*	
Vividness	0.28 (0.05)	6.14	< 0.001*		0.12 (0.04)	2.60	0.01*	

*β* standardised beta coefficient, *SE* standard error

^1^Model 1 controlled for age and gender

^2^Model 2 controlled for age, gender, and Time 1 depressive symptoms

*Indicates *p* < 0.05

**Table 6:** Results of multiple regression analysis for vividness predicting Time 2 social anxiety symptoms (*N* = 616)

Variable	Model 1^1^				Model 2^2^			
	β (SE)	t	*p*	*R*^2^	β (SE)	t	*p*	*R*^2^
*Step 1*				0.55				0.56
Age	− 0.01 (0.03)	− 0.14	0.88		− 0.02 (0.03)	− 0.44	0.66	
Gender (male)	− 0.08 (0.06)	− 1.31	0.19		− 0.03 (0.06)	− 0.50	0.61	
Social anxiety	0.74 (0.03)	23.78	< 0.001*		0.66 (0.04)	17.55	< 0.001*	
Depression					0.15 (0.04)	3.47	< 0.001*	
*Step 2*				0.56				0.58
Age	− 0.01 (0.03)	− 0.16	0.87		− 0.01 (0.03)	− 0.44	0.66	
Gender (male)	− 0.08 (0.06)	− 1.31	0.19		− 0.03 (0.06)	− 0.50	0.62	
Social anxiety	0.73 (0.03)	22.07	< 0.001*		0.66 (0.04)	17.90	< 0.001*	
Depression					0.16 (0.05)	3.25	0.01*	
Vividness	0.01 (0.04)	0.31	0.08		− 0.02 (0.04)	− 0.36	0.72	

*β* standardised beta coefficient, *SE* standard error

^1^Model 1 controlled for age, gender, and Time 1 social anxiety symptoms

^2^Model 2 controlled for age, gender, Time 1 social anxiety symptoms, and Time 1 depressive symptoms

*Indicates *p* < 0.05

### Topic Modelling of Negative Self-images

**Fig. 1:**
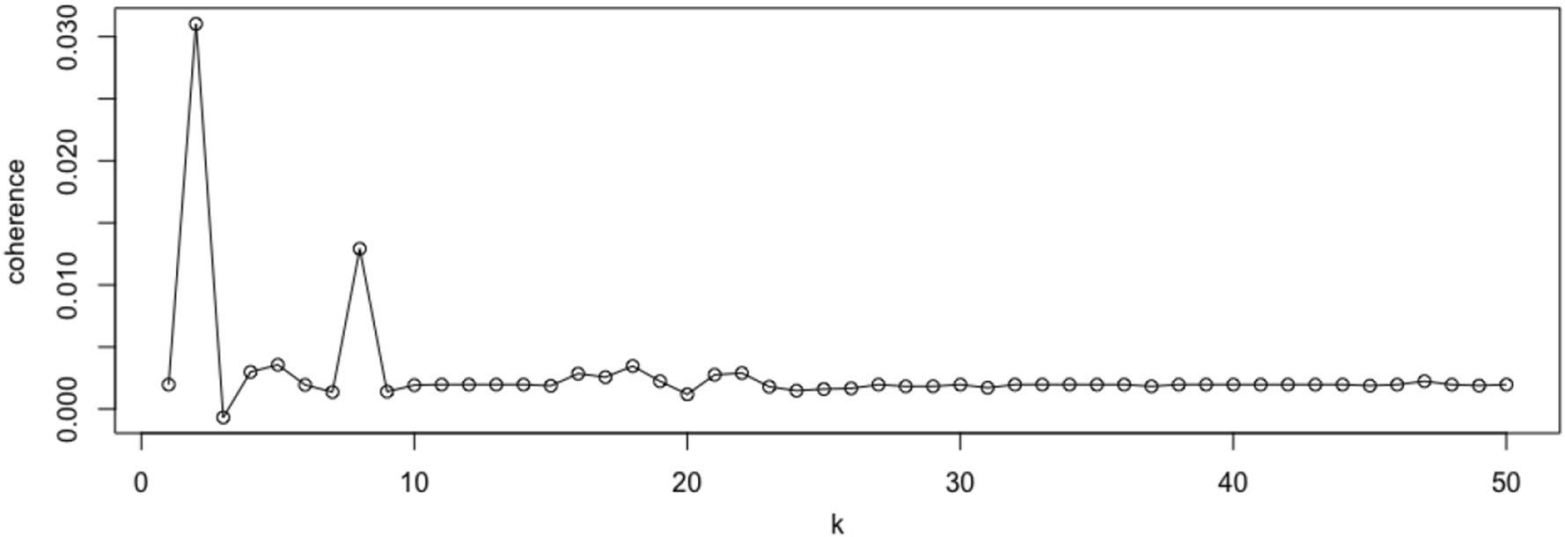
Number of topics (k) versus average coherence value (coherence)

**Fig. 2:**
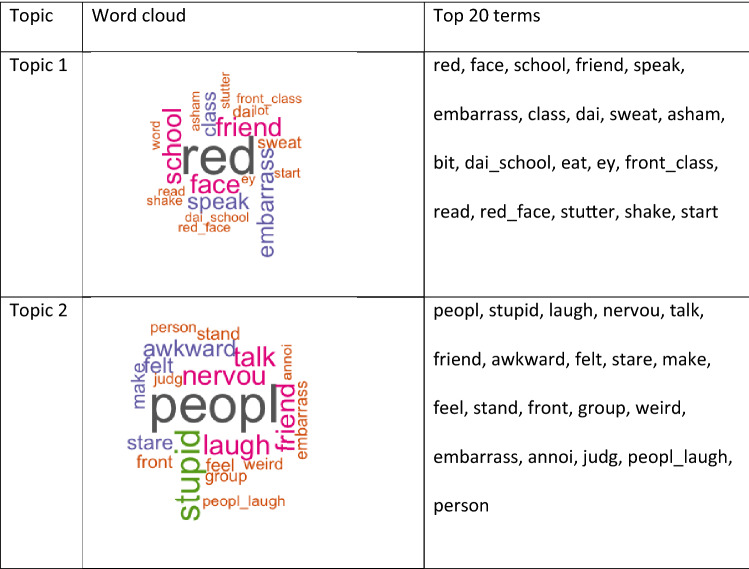
Word clouds and the top 20 terms for topic 1 and topic 2. Each word cloud displayed the top 20 terms. The size of each word was based on the posterior probability of a word given a topic. All words were reduced to their word stem (e.g. ‘day’ and ‘days’ were converted to ‘dai’, ‘eye’ and ‘eyes’ were converted to ‘ey’)

Figure 1 showed the number of topics versus average coherence value (coherence). A higher average coherence value reflects better topic quality. The average coherence value peaked when there were two topics. Based on this result, we present the word clouds of these two topics (Fig. 2). The top terms of Topic 1 were related to the fear of appearing anxious and the visible signs of anxiety (e.g., red face, sweat, stutter, shake). The top terms of Topic 2 were related to the fear of being judged or being seen as unacceptable (e.g., stupid, laugh, awkward, weird). Topic 2 has a higher topic prevalence than Topic 1 (69.23% versus 30.72%), meaning that nearly one-third of the participants reported self-images related to the fear of being judged or being seen as unacceptable.

## Discussion

The aim of the current study was to examine the concurrent and prospective associations between adolescent social anxiety and two image characteristics, namely, observer-perspective and vividness. Both image characteristics significantly predicted concurrent social anxiety symptoms beyond the influence of age and gender. Furthermore, observer-perspective, but not vividness, significantly predicted prospective levels of social anxiety symptoms beyond the influence of age, gender, baseline social anxiety symptoms, and baseline depressive symptoms. These findings suggest self-images experienced from an observer perspective may be implicated in the maintenance of social anxiety in adolescents. Another aim of this study was to understand the common themes of negative self-imagery associated with social anxiety in adolescents. Using topic modelling, we found that these negative self-images are related to the fear of appearing anxious and the fear of being judged or viewed as unacceptable. These findings suggest certain content of negative imagery may be particularly relevant to adolescent social anxiety and may be usefully targeted in psychological interventions.

Extending the findings of cross-sectional studies (Alfano et al., 2008; Leigh et al., 2020; Schreiber & Steil, 2013), this study provides evidence that the observer-perspective of negative self-imagery is prospectively related to adolescent social anxiety symptoms. This finding suggests adolescents who imagine seeing themselves from other people’s perspective in a social situation tend to experience higher levels of social anxiety symptoms over time. This may be because images of oneself seen from the ‘outside-in’ may be more plausible and so the individual is then more liable to use the image as evidence for how they really come across to others. Unfortunately, though, as the image is distorted and excessively negative, the individual is left believing more strongly that they come across poorly and feeling more anxious (Leigh et al., 2020). Observer-perspective continued to be associated with social anxiety prospectively over and above baseline depression symptoms. It therefore appears that although mental imagery is common to both depression and social anxiety, an observer-perspective is independently associated with social anxiety symptoms over time. The increase in variance in outcome social anxiety explained by observer perspective is small (1%). However, it is worth pointing out that small effects can accumulate over time (Funder & Ozer, 2019). So, the tendency to engage in observer perspective imagery may affect how socially anxious you feel only modestly, but this could accumulate relatively quickly over time, leading to greater effects in the long-term. In the present study we replicated the reported concurrent association between image vividness and social anxiety symptoms in adolescents [see Schreiber et al. (2012) for a review]. Interestingly we observed a non-significant effect of image vividness on prospective social anxiety symptoms. If replicated, the finding suggests that vividness is a correlate or consequence of social anxiety. If the current findings are replicated with a clinical sample, it would have implications for optimising therapeutic interventions. In particular, they suggest imagery valence and observer-perspective should be targeted. Whilst vividness is not indicated as a therapeutic target, its reduction could perhaps be considered as evidence of a successful imagery intervention.

Using topic modelling we identified two latent topics of negative self-imagery associated with adolescent social anxiety, namely the fear of appearing anxious and the fear of being judged. This two-topic solution had the highest topic quality as reflected by the coherence value and is broadly comparable to the two themes of social anxiety related imagery reported in a qualitative study with adults (Dobinson et al., 2020). Interestingly, the two topics also parallel the two groups of cognitions we recently found to be commonly reported by socially anxious adolescents, one related to negative self-concept (e.g., fear of being judged, being unlikeable) and fear of appearing anxious (e.g. fear of blushing, fear of sweating) (Leigh & Clark, 2021a, b). The images can be thought to be the sensory representations of these verbal cognitions (Holmes & Mathews, 2010).

This study is the first prospective study to examine the role of image characteristics in predicting prospective social anxiety symptoms in adolescents. Our study offers evidence that observer-perspective may be implicated in the maintenance of social anxiety in adolescents. This provides a good theoretical foundation for conducting experimental research in the future, for example, manipulating observer-perspective of imagery during a social task. The use of LDA enables researchers to discover latent themes of negative-self imagery from highly unstructured verbal data. This type of analysis does not require researchers to infer topics from each data point subjectively, and therefore, reduces interpretative bias and manual errors (Abram et al., 2020). In addition, this analytic approach uses a transparent and time-efficient way to process verbal data, this ensures semantic content and relationships are considered statistically.

Despite these strengths, several limitations of this study should be noted. First, the present study used single-item measures to assess vividness and perspective-taking, which may subject to random measurement errors. Although our study asked participants to rate how clear or vivid the mental image is for them, we did not assess the amount of detail in these mental images, such as one’s facial expressions or behaviours in the image. Second, the use of self-report measures may increase shared method variance, leading to an inflation of correlations between the variables measured. Third, although the present study used a quantitative method to identify latent topics, this is not a deterministic method. For example, the number of topics may change when the study sample size increases. Fourth, although the present study sample included a fairly large community sample, our findings may not generalise to adolescents who have a clinical diagnosis of SAD. Fifth, we used a validated questionnaire to elicit self-imagery and the associated characteristics, but it is possible that giving participants specific instructions to describe their self-images may restrict the range of themes reported. In future studies it may be helpful to use an alternative more open-ended method to elicit negative self-imagery, for example, the semi-structured interview method of Hackmann et al. (2000). Sixth, while the analyses support the suggestion of a longitudinal association between observer perspective negative imagery and later social anxiety, it does not necessarily indicate that changes in observer perspective imagery lead to changes in social anxiety. Due to the lack of imagery measures at outcome, we cannot rule out the possibility that the longitudinal association observed is a result of a combination of concurrent associations and high stability in observer perspective imagery and social anxiety. Future studies using a cross-lagged design could test this possibility. Finally, the results should be interpreted with caution as we did not adjust for multiple comparisons.

This study has some important implications for the prevention and early intervention of SAD. Supporting young people to develop accurate self-images may protect them against the development of social anxiety symptoms. Imagery techniques such as video feedback (Warnock-Parkes et al., 2017) may be helpful for adolescents with SAD, to discover that the self-images they experience are distorted and excessively negative and to develop more positive and accurate self-images.

## Supplementary Information

Below is the link to the electronic supplementary material.

Supplementary file1 (DOCX 224 KB)

## Declarations

### Research Involving Human and Animal Participants

Ethical approval for the study was obtained from University of Oxford Central University Research Ethics Committee (R54283/RE001) and all procedures were in accordance with the ethical standards of the institutional research committee and with the 1964 Declaration of Helsinki and its later amendments or comparable ethical standards.
