# Supplementary material for: Characterising Negative Mental Imagery in Adolescent Social Anxiety
Source: Cognit Ther Res. 2022 Jul 5;46(5):956–66. doi: 10.1007/s10608-022-10316-x (PMC9492563; doi:10.1007/s10608-022-10316-x)
Supplement: Supplementary file 1 — Supplementary file1 (DOCX 224 KB) [file 10608_2022_10316_MOESM1_ESM.docx]

**Appendix**

Appendix A1. Questionnaire of Recurrent Images in Social Phobia (QRI-SP)

| Questionnaire of Recurrent Images in Social Phobia (QRI-SP) | | | | | | | | | | |
| --- | --- | --- | --- | --- | --- | --- | --- | --- | --- | --- |
| Sometimes we see images going through our minds when we feel very anxious. These images are like photos or film clips. Often, these images are about ourselves (called self-images). These self-images can seem strange. We would like to know about the self-images you have in certain situations. Try to think of a social situation in the past, either at school or with friends, when you felt that you looked awkward, foolish, or frightened. Concentrate on this self-image in as much detail as possible. Then, please answer the following questions: | | | | | | | | | | |
| 1. Please describe this image in a few words. | | | | | | | | | | |
|  | | | | | | | | | | |
| 1. Please insert a cross mark (X) on the line:   In this image, … | | | Not at all | | | | Extremely | | | |
| 1. … I get into a situation in which I am judged negatively by others. | | | 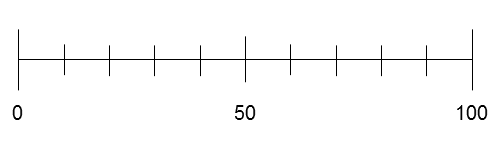 | | | | | | | |
| 1. … I make a mistake and other people notice. | | | 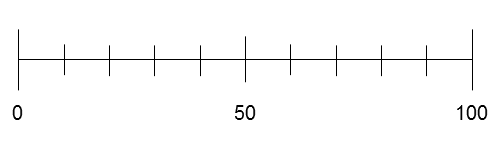 | | | | | | | |
| 1. … other people notice my anxiety and insecurity. | | | 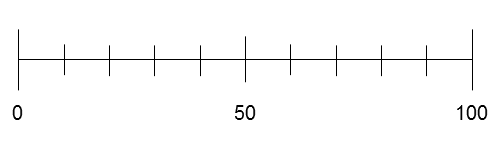 | | | | | | | |
| 1. … I worry a lot that someone may notice I am anxious. | | | 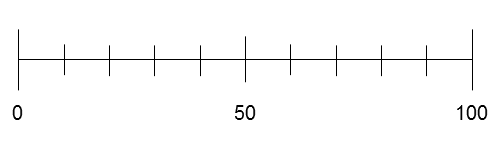 | | | | | | | |
| 1. … I feel nervous and insecure when I am in a public place. | | | 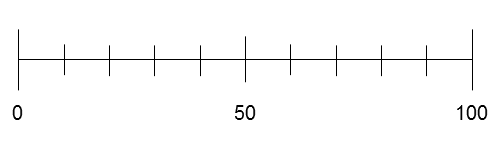 | | | | | | | |
| 1. … I think others are not interested in me at all. | | | 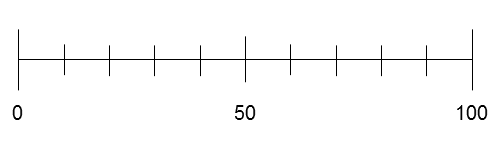 | | | | | | | |
| 1. … my worst fear has come true. | | | 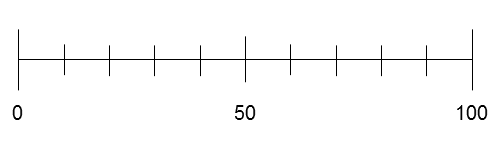 | | | | | | | |
| 1. … I performed poorly. | | | 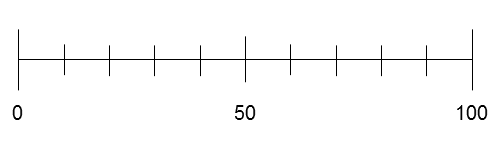 | | | | | | | |
| 1. Which feelings do you experience in this image? I feel: | | | Not at all | | | | Extremely | | | |
| 1. … shame. | | | 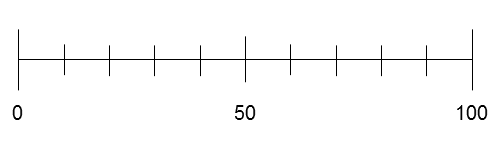 | | | | | | | |
| 1. … anxious. | | 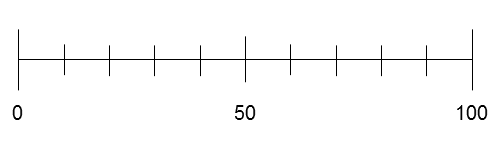 | | | | | | | | |
| 1. … embarrassed. | | 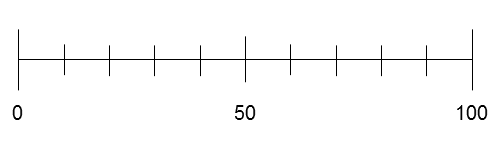 | | | | | | | | |
| 1. … angry. | | 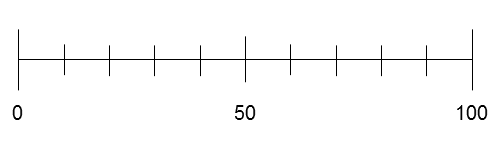 | | | | | | | | |
| 1. … stupid. | | 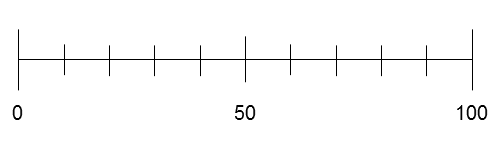 | | | | | | | | |
| 1. … insecure. | | 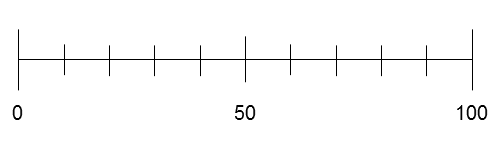 | | | | | | | | |
| 1. … nervous. | | 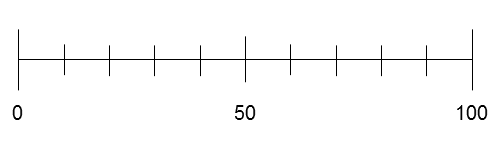 | | | | | | | | |
| 1. … vulnerable. | | 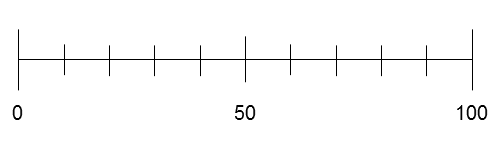 | | | | | | | | |
| 1. … humiliated/ ridiculous. | | 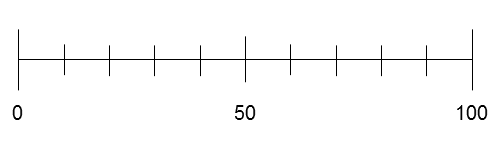 | | | | | | | | |
| 1. … happy. | | 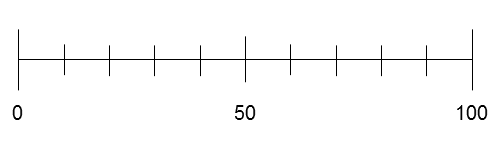 | | | | | | | | |
| 1. … Others: ______________ . | | 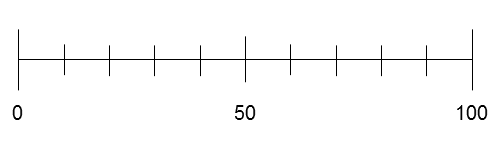 | | | | | | | | |
| 1. How bad is this image for you? | | 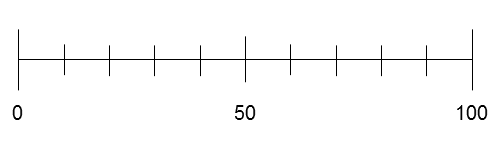 | | | | | | | | |
| 1. How clear/ vivid is this image for you? | | 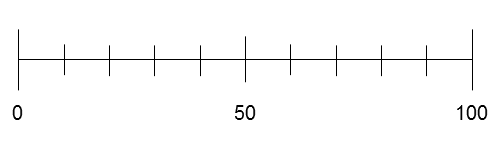 | | | | | | | | |
|  |  | Not vivid /  Clear at all | | | | | | Very vivid /  Clear | | |
| 1. How often did you have such image in difficult social situations over the last 14 days? | | Never | | Seldom | | Some-times | | | Often | Always |
|  |  | □ | | □ | | □ | | | □ | □ |
| 1. What do you notice about your image?   Please tick what applies to your image. | | | | | | | | | | |
| 1. I can see something. | | □ | | □ | | □ | | | □ | □ |
| 1. I can hear something. | | □ | | □ | | □ | | | □ | □ |
| 1. I taste something. | | □ | | □ | | □ | | | □ | □ |
| 1. I smell something. | | □ | | □ | | □ | | | □ | □ |
| 1. I notice changes in my body, e.g., faster heartbeat | | □ | | □ | | □ | | | □ | □ |
| 1. Please concentrate on your image again. Which perspective do you take in it? | | | | | | | | | | |
| In my image, I see myself and the things around me through my eyes (field perspective). | 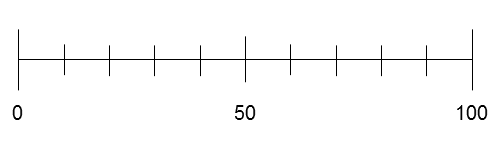 | | | | In my image, I see myself and the things around me through other people’s eyes (observer-perspective). | | | | | |

| 1. Have you ever experienced a similar situation like the one from your image in real life? | □ yes | □ no | |
| --- | --- | --- | --- |
| 1. If yes: Please answer the following questions. Please describe this memory in a few words. If no: This is the end of the questionnaire. | | | |
|  | | | |
| 1. How old were you in this memory? | ________________ | | |
| 1. How bad is this memory for you? | 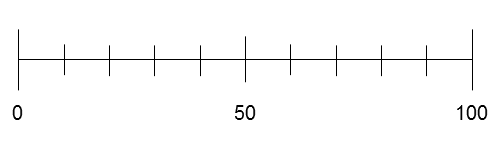 | | |
|  | not at all | extremely | |
| 1. How clear/ vivid is this memory for you? | 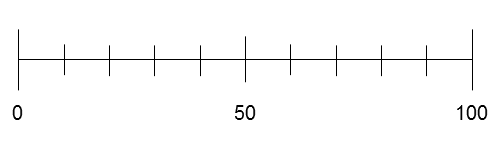 | | |
|  | not vivid /  clear at all | | very vivid /  clear |

Appendix A2: Results of multiple regression analyses using complete case to handle missing data

| **Table 1.** Results of multiple regression analysis for observer-perspective predicting Time 1 social anxiety symptoms (*N* = 330) | | | | | | | | |
| --- | --- | --- | --- | --- | --- | --- | --- | --- |
|  | Model 1^1^ | | |  | Model 2^2^ | | |  |
| Variable | β (SE) | t | *p* | *R*^2^ | β (SE) | t | *p* | *R*^2^ |
| **Step 1** |  |  |  | **0.10** |  |  |  | **0.37** |
| Age | 0.13 (0.05) | 2.66 | .008* |  | 0.07 (0.04) | 1.60 | .111 |  |
| Gender (Male) | -0.55 (0.11) | -5.23 | < .001* |  | -0.18 (0.09) | -1.93 | .06 |  |
| Depression |  |  |  |  | 0.56 (0.05) | 11.77 | < .001* |  |
| **Step 2** |  |  |  | **0.17** |  |  |  | **0.38** |
| Age | 0.10 (0.05) | 2.02 | .04* |  | 0.07 (0.04) | 1.62 | .11 |  |
| Gender (Male) | -0.54 (0.10) | -5.34 | < .001* |  | -0.19 (0.09) | -2.03 | .04* |  |
| Depression |  |  |  |  | 0.51 (0.05) | 9.99 | < .001* |  |
| Observer-perspective | 0.28 (0.05) | 5.45 | < .001* |  | 0.12 (0.05) | 2.56 | .01* |  |

*Note.* β *=* standardised beta coefficient; SE = standard error. ^1^ Model 1 controlled for age and gender. ^2^ Model 2 controlled for age, gender, and Time 1 depressive symptoms. * indicates *p* < .05.

| **Table 2.** Results of multiple regression analysis for vividness predicting Time 1 social anxiety symptoms (*N* = 330) | | | | | | | | |
| --- | --- | --- | --- | --- | --- | --- | --- | --- |
|  | Model 1^1^ | | |  | Model 2^2^ | | |  |
| Variable | β (SE) | t | *p* | *R*^2^ | β (SE) | t | *p* | *R*^2^ |
| **Step 1** |  |  |  | **0.10** |  |  |  | **0.37** |
| Age | 0.14 (0.05) | 2.66 | 0.01* |  | 0.07 (0.04) | 1.60 | 0.11 |  |
| Gender (Male) | -0.55 (0.11) | -5.23 | < .001* |  | -0.18 (0.09) | -1.93 | 0.06 |  |
| Depression |  |  |  |  | 0.56 (0.05) | 11.77 | < .001* |  |
| **Step 2** |  |  |  | **0.19** |  |  |  | **0.38** |
| Age | 0.13 (0.05) | 2.51 | .01* |  | 0.06 (0.04) | 1.28 | .20 |  |
| Gender (Male) | -0.50 (0.10) | -4.86 | < .001* |  | -0.20 (0.09) | -2.19 | .03* |  |
| Depression |  |  |  |  | 0.51 (0.05) | 10.64 | < .001* |  |
| Vividness | 0.30 (0.05) | 6.03 | < .001* |  | 0.15 (0.05) | 3.25 | .001* |  |

*Note.* β *=* standardised beta coefficient; SE = standard error. ^1^ Model 1 controlled for age and gender. ^2^ Model 2 controlled for age, gender, and Time 1 depressive symptoms. * indicates *p* < .05.

| **Table 3.** Results of multiple regression analysis for observer-perspective predicting Time 2 social anxiety symptoms (*N* = 240) | | | | | | | | | | | |
| --- | --- | --- | --- | --- | --- | --- | --- | --- | --- | --- | --- |
|  | Model 1^1^ | | |  | | Model 2^2^ | | | | |  |
| Variable | β (SE) | t | *p* | | *R*^2^ | | β (SE) | t | *p* | *R*^2^ | |
| **Step 1** |  |  |  | | **0.55** | |  |  |  | **0.56** | |
| Age | -0.01 (0.04) | -0.18 | .85 | |  | | -0.01 (0.04) | -0.29 | .77 |  | |
| Gender (Male) | -0.11 (0.09) | -1.16 | .25 | |  | | -0.04 (0.09) | -0.41 | .68 |  | |
| Social Anxiety | 0.73 (0.05) | 15.73 | < .001* | |  | | 0.69 (0.05) | 12.83 | < .001* |  | |
| Depression |  |  |  | |  | | 0.15 (0.05) | 2.95 | .003* |  | |
| **Step 2** |  |  |  | | **0.56** | |  |  |  | **0.57** | |
| Age | -0.01 (0.04) | -0.27 | .79 | |  | | -0.02 (0.04) | -0.35 | .72 |  | |
| Gender (Male) | -0.12 (0.09) | -1.30 | .20 | |  | | -0.05 (0.09) | -0.57 | .57 |  | |
| Social Anxiety | 0.70 (0.05) | 14.81 | < .001* | |  | | 0.64 (0.05) | 12.40 | < .001* |  | |
| Depression |  |  |  | |  | | 0.14 (0.05) | 2.73 | .01* |  | |
| Observer-perspective | 0.09 (0.04) | 2.01 | .045* | |  | | 0.07 (0.04) | 1.69 | .09 |  | |

*Note.* β *=* standardised beta coefficient; SE = standard error. ^1^ Model 1 controlled for age, gender, and Time 1 social anxiety symptoms. ^2^ Model 2 controlled for age, gender, Time 1 social anxiety symptoms, and Time 1 depressive symptoms. * indicates *p* < .05.

| **Table 4.** Results of multiple regression analysis for vividness predicting Time 2 social anxiety symptoms (*N* = 240) | | | | | | | | |
| --- | --- | --- | --- | --- | --- | --- | --- | --- |
|  | Model 1^1^ | | |  | Model 2^2^ | | |  |
| Variable | β (SE) | t | *p* | *R*^2^ | β (SE) | t | *p* | *R*^2^ |
| **Step 1** |  |  |  | 0.55 |  |  |  | 0.56 |
| Age | -0.01 (0.04) | -0.18 | .85 |  | -0.01 (0.04) | -0.29 | .77 |  |
| Gender (Male) | -0.11 (0.09) | -1.16 | .25 |  | -0.04 (0.09) | -0.41 | .68 |  |
| Social Anxiety | 0.73 (0.05) | 15.74 | < .001* |  | 0.66 (0.05) | 12.83 | < .001* |  |
| Depression |  |  |  |  | 0.15 (0.05) | 2.95 | .003* |  |
| **Step 2** |  |  |  | 0.55 |  |  |  | 0.56 |
| Age | -0.01 (0.04) | -0.18 | .86 |  | -0.01 (0.04) | -0.29 | .77 |  |
| Gender (Male) | -0.11 (0.09) | -1.17 | .24 |  | -0.04 (0.09) | -0.39 | .70 |  |
| Social Anxiety | 0.72 (0.05) | 14.94 | < .001* |  | 0.66 (0.05) | 12.73 | < .001* |  |
| Depression |  |  |  |  | 0.16 (0.05) | 2.89 | .004* |  |
| Vividness | 0.03 (0.05) | 0.60 | .55 |  | -0.01 (0.05) | -0.23 | .82 |  |

*Note.* β *=* standardised beta coefficient; SE = standard error. ^1^ Model 1 controlled for age, gender, and Time 1 social anxiety symptoms. ^2^ Model 2 controlled for age, gender, Time 1 social anxiety symptoms, and Time 1 depressive symptoms. * indicates *p* < .05.
